# Complete Genome Sequences of Mycobacteriophages HarryOW and Peeb

**DOI:** 10.1128/MRA.00112-21

**Published:** 2021-04-08

**Authors:** Fernando E. Nieto-Fernandez, Christos Noutsos, John Kleopoulos, Olubusola Babalola, Belle L. Connaught, Balquees Shafique, Shannon Farnum, Hammad Nawaz, Raymond Catapano, Rita M. Reddy, Jorge Morales, Patricia Roccanova, Shirley Barrera

**Affiliations:** a Department of Biological Sciences, SUNY Old Westbury, Old Westbury, New York, USA; b SUNY OW Science and Technology Entry Program, Old Westbury, New York, USA; c Westbury High School, Old Westbury, New York, USA; d The City College of New York, New York, New York, USA; Queens College CUNY

## Abstract

HarryOW and Peeb are Mycobacterium smegmatis mc^2^ 155 *Siphoviridae* temperate phages with 52,935 and 41,876 base pairs in genome length, respectively. HarryOW belongs to the A1 subcluster and Peeb to the G1 subcluster. They were isolated and annotated by students from the SUNY Old Westbury Science and Technology Entry Program.

## ANNOUNCEMENT

**FIG 1:**
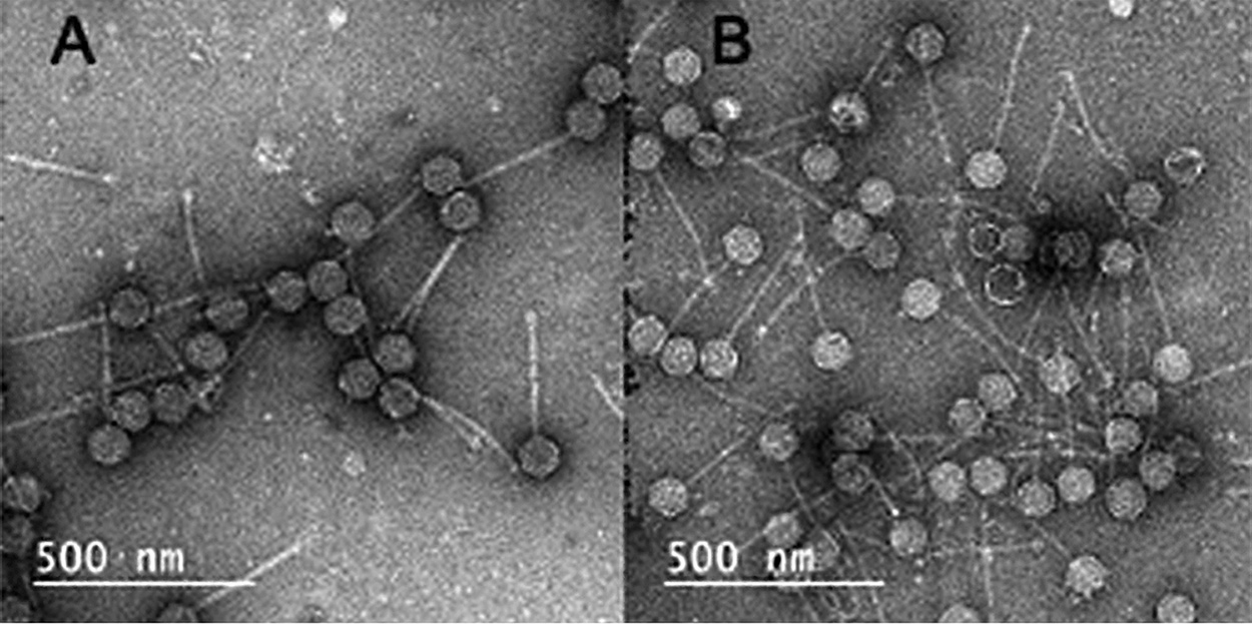
TEM samples mounted on Formvar-coated copper grids stained with uranyl acetate and imaged with a JEOL JEM-2100 at ×20,000. (A) HarryOW, icosahedral capsid (∅ = 103 nm, tail = 283 nm). (B) Peeb, icosahedral capsid (∅ = 93 nm, tail = 304 nm).

HarryOW and Peeb were isolated from soils located in Westbury, New York (40.760026 N, 73.589 W). Isolation was done by enrichment with Mycobacterium smegmatis mc^2^ 155 following two cycles of purification and amplification in 7H9 top agar at 37°C (1). Phages were collected from a high-titer lysate by high-speed centrifugation; mounted on carbon-stabilized, Formvar-coated copper transmission electron microscopy (TEM) grids stained with uranyl acetate; and imaged with a JEOL JEM-2100 TEM (Fig. 1) (1). DNA was extracted using a Promega DNA Wizard kit with a modified protocol (1). Sequencing was done at the North Carolina State University Genomic Science Laboratory. Genomic libraries were prepared using an Illumina Truseq Nano DNA library kit. Pooled libraries were run on an Illumina MiSeq system yielding 670,230 and 661,512 single-end 150-base pair (bp) reads with average coverages of 1,783× and 2,189× for HarryOW and Peeb, respectively (Table 1). The raw reads were assembled using Newbler and checked for completeness and genomic termini with Consed at the University of Pittsburgh (2).

**TABLE 1:** Genome features and accession numbers for HarryOW and Peeb

Phage name	GenBank accession no.	SRA accession no.	Avg coverage (×)	Cluster	Genome length (bp)	GC content (%)	No. of genes
HarryOW	MT776812	SRX10050390	1,783	A1	52,935	63.8	94
Peeb	MW291028	SRX10050392	2,189	G1	41,876	66.6	62

Their genomes were annotated using DNA Master (http://cobamide2.bio.pitt.edu/computer.htm), and gene prediction tools GLIMMER v3.0 (3), GeneMark v2.5 (4), and Starterator v1.1 were used to determine gene start sites. tRNA and transfer-messenger RNA (tmRNA) predictions were made using ARAGORN v1.2.38 (5) and tRNAscan-SE v3.0 (6). Functional assignments were made using BLAST v2.9 (7), HHpred (8), and Phamerator (9). Manual annotation was done in parallel with PECAAN (10). Default settings were used for all programs. Phages in a cluster share sequence similarity over 50% of their genome length (11).

Peeb is a G1 cluster phage with 41,876 base pairs (bp), a GC content of 66.6%, 62 genes, and an 11-bp 3′ sticky overhang terminus. A total of 23 of the 62 genes are of known function. Peeb is 99% genetically identical to Schiebel (GenBank accession no. MH045569) with whom it shares 94.32% gene content and 58 gene phams. The integration cassette genes of Peeb are SEA_PEEB_32, a tyrosine integrase; SEA_PEEB_33, an immunity repressor; and SEA_PEEB_34, an excise. SEA_PEEB_32 and SEA_PEEB_33 are the only two genes in the Peeb genome coded on the reverse strand. SEA_PEEB_54 is a mycobacteriophage mobile element 1 (MPME1) found in other members of this gene pham and of this cluster (12).

HarryOW is an A1 cluster mycobacteriophage with 52,935 bp, a GC content of 63.8%, 94 genes, and an 11-bp 3′ sticky overhang terminus. A total of 35 of the 94 genes are of known function. Forty genes are on the forward strand, mostly on the left arm of the genome, except for genes SEA_HARRYOW_90, SEA_HARRYOW_91, and SEA_HARRYOW_93. HarryOW is 98% genetically identical to Rutherferd (GenBank accession no. NC_052461), sharing 80.02% gene content similarity and 74 phams. As in many of the A cluster phages, the immunity repressor SEA_HARRYOW_76 is not located adjacent to the serine integrase SEA_HARRYOW_37, and the DNA primase has two overlapping reading frames, namely, SEA_HARRYOW_54 and SEA_HARRYOW_55. One of the three minor tail protein genes, SEA_HARRYOW_6, is located in the left arm of the genome, upstream of the lysin A SEA_HARRYOW_10, lysin B SEA_HARRYOW_11, and the terminase SEA_HARRYOW_12. The other two minor tail protein genes, namely, SEA_HARRYOW_26 and SEA_HARRYOW_28, are located after the tape measure gene SEA_HARRYOW_25.

### Data availability.

The genome sequences for Peeb and HarryOW have been deposited in GenBank and the Sequence Read Archive (see Table 1).
